# Translation, Cultural Adaptation, and Content Validity of a Modified Italian Version of the Jackson/Cubbin Pressure Injury Risk Assessment Scale for ICU Patients

**DOI:** 10.3390/nursrep15070256

**Published:** 2025-07-14

**Authors:** Chiara Rollo, Daniela Magnani, Sara Alberti, Brigitta Fazzini, Sergio Rovesti, Paola Ferri

**Affiliations:** 1 Territorial Emergency Nurse, Azienda Sanitaria Locale, 72100 Brindisi, Italy; chiara.rollo@hotmail.it; 2 Intensive Unit, University Hospital of Modena, 41124 Modena, Italy; magnani.daniela@aou.mo.it; 3 Clinical and Experimental Medicine PhD Program, Department of Biomedical, Metabolic and Neural Sciences, University of Modena and Reggio Emilia, 41121 Modena, Italy; sara.alberti@unimore.it; 4 Department of Medicine, ASST-Franciacorta, 25049 Brescia, Italy; 5 William Harvey Research Institute, Barts and The London School of Medicine & Dentistry, Queen Mary University of London, London E1 2AT, UK; 6 Intensive Care Medicine, Royal London Hospital, Barts Health NHS Trust, London E1 1BB, UK; 7 Department of Biomedical, Metabolic and Neural Sciences, University of Modena and Reggio Emilia, 41100 Modena, Italy; sergio.rovesti@unimore.it (S.R.); paola.ferri@unimore.it (P.F.)

**Keywords:** pressure ulcer, risk assessment, intensive care units, translation, cultural adaptation, Jackson/Cubbin

## Abstract

**Background/Objectives**: The Jackson/Cubbin scale is a recommended tool to assess the risk of pressure injury in intensive care unit (ICU) patients. This scale is deemed to have superior predictive validity compared to the Braden scale. Many Italian nurses struggle with reading and applying the tool in English. This language barrier results in a lack of use of the Jackson/Cubbin scale clinically, meaning that patients potentially experience worse outcomes. This study aims to translate the original English version of the Jackson/Cubbin scale into the Italian language, conduct a cultural adaptation, and verify its content validity. **Methods**: An observational study was conducted using Beaton’s five-step methodology: (1) forward translation, (2) synthesis, (3) back-translation, (4) expert committee approval using Fleiss’ Kappa (κ) index, and (5) pre-testing, where participants assessed item clarity on a dichotomous scale (clear/unclear). Items deemed unclear by 20% or more of the sample were revised. Content validity was assessed using the Content Validity Index (CVI). **Results**: Fleiss’ κ index was 0.74. Item 3 “PMH-affecting condition” was unclear to 36% of the sample and required revision. The item-level CVI (I-CVI) was >0.78 for each item. The scale-level CVI (S-CVI) and the scale-level CVI using the average method (S-CVI-Ave) were 0.92 and 0.94, respectively. **Conclusions**: The translation process resulted in a linguistically accurate scale requiring content modifications to reflect current evidence and reduce inter-rater variability. This may improve implementation of the Jackson/Cubbin scale in clinical practice for Italian nurses and reduce the incidence of pressure injury for ICU patients.

## 1. Introduction

Pressure injuries represent a serious complication for patients admitted to intensive care units (ICUs). The DecubICUs study reported a 26.6% prevalence of pressure injuries in this setting [1]. ICU patients are at increased risk of developing pressure injuries due to multiple predisposing factors [2,3,4], including prolonged immobility [2,3], advanced age [2,3,5], diabetes mellitus [2,4], hypotension [2,3], ICU length of stay [2,4,5], mechanical ventilation [2,3,4,5], and vasopressor use [2,3,4]. In addition to these factors, the use of medical devices [6,7] contributes to the development of “medical device-related pressure injuries”. The development of pressure injuries leads to negative outcomes for both patients and healthcare systems, including reduced quality of life [8], prolonged length of stay [9], and increased mortality and morbidity [10,11] leading to higher cost-spending for healthcare systems [12]. Determining the risk of developing pressure injuries is a fundamental aspect of preventive care. For this reason, the European Pressure Ulcer Advisory Panel (EPUAP), the National Pressure Injury Advisory Panel (NPIAP), and the Pan Pacific Pressure Injury Alliance (PPPIA) recommend the use of a validated screening tool [4]. Currently, approximately 40 risk assessment scales for pressure injuries are reported in the literature. The most commonly used scoring systems are the Braden, the Norton, and the Waterlow scale. In the ICU setting, the Braden scale is the most widely adopted [13,14]. However, this is not specifically designed for critically ill patients, and it has demonstrated limited predictive validity and a tendency to overestimate risk [15,16].

In fact, a large meta-analysis assessing 10,044 critically ill patients showed that the Braden scale has a high diagnostic value (DOR: 6.28) but poor discriminative performance (area under the curve: 0.78), especially for specificity (specificity: 28%, sensitivity: 89%) [17]. A subsequent review highlighted inconsistency of its psychometric properties, with substantial variability in terms of reliability, validity, and cut-off score diagnostic value (sensitivity: 41–100%, specificity: 5–79%, positive predictive value: 14–85%, negative predictive value: 38–100%, and area under the curve: 29–86%) suggesting that the scale should be used cautiously as a predictive tool in ICU settings [18].

To improve predictive validity, more specific risk assessment tools for ICU settings have been analysed in the literature (e.g., CALCULATE, COMHON Index, and Jackson/Cubbin), revealing better results in most cases [19,20]. Specifically, recent studies have focused on the psychometric properties of the Jackson/Cubbin scale [21,22], reporting good diagnostic and discriminative values (sensitivity: 0.81, specificity: 0.76, positive likelihood ratio: 3.34, negative likelihood ratio: 0.25, DOR: 13.24, and ROC AUC: 0.84) [23]. This suggests the superiority of the Jackson/Cubbin scale in predicting pressure injury risk in ICU patients (i.e., patients at risk and those not at risk), with a smaller statistical gap between sensitivity and specificity and a superior discriminatory performance compared to the Braden scale [17].

A recent umbrella review conducted by Hillier and colleagues (2025) further confirmed the superior predictive performance of the Jackson/Cubbin scale compared to other ICU-specific tools, such as the EVARUCI and the Braden scales. In studies comparing ICU-specific tools to the Braden scale, the Jackson/Cubbin scale showed strong convergent validity (r = 0.80) compared to the COMHON Index (r = 0.70) the Norton scale (r = 0.77), and the Waterlow scale, which demonstrated inconsistent values (r = 0.22–0.72). These findings reinforce the psychometric strength and applicability of the Jackson/Cubbin scale in the ICU setting [24].

Furthermore, the Jackson/Cubbin scale includes clinical parameters relevant to critical care such as vasopressor use, haemodynamic stability, and oxygenation, which are not addressed in generalist tools like the Braden scale. This clinical specificity makes it particularly suited for ICU patients [24]. A recent multi-centre study comparing the Waterlow and Jackson/Cubbin scales highlighted the superior predictive performance of the Jackson/Cubbin scale, particularly in ICU settings where clinical complexity requires more tailored assessment tools [25]. Although the Jackson/Cubbin scale demonstrates superior predictive validity, its implementation and effective use in routine nursing practice remain limited, particularly in non-English-speaking countries. This may be due to the lack of a culturally and linguistically adapted version, creating a barrier to its widespread adoption. For this reason, the translation and cultural adaptation of the Jackson/Cubbin scale represent crucial steps not only for its practical application in diverse healthcare contexts but also to enable rigorous comparative research with other risk assessment tools.

Our work aims to conduct a translation and cultural adaptation study of the Jackson/Cubbin scale into Italian, alongside a content validity verification to understand how to effectively implement the scale, to overcome barriers to its use. This approach aligns with the findings from a recent systematic review analysing the psychometric properties of the Braden scale in ICU settings [18]. This review highlighted variable evidence on its reliability and validity, with limited studies on inter-rater reliability and internal consistency. Therefore, the authors recommended the need for further comparative studies including ICU-specific tools (i.e., the Jackson/Cubbin scale) to determine the most appropriate risk assessment tool for ICU patients [18]. Our study aims to take the first step toward the broader psychometric validation of the Jackson/Cubbin scale in the Italian context, paving the way for future studies aiming to test its predictive validity, inter-rater reliability, and clinical utility.

## 2. Materials and Methods

### 2.1. Study Design and Setting

This study is designed as an observational and descriptive cross-sectional study. The study was conducted from April 2024 to July 2024 across two intensive care units in the University Hospital of Modena (Italy). The study commenced following approval from the Management Board of the University Hospital of Modena and the Ethics Committee.

We followed the STROBE checklist to ensure clear and transparent reporting of the observational study findings.

### 2.2. Measurement Instrument

The Jackson/Cubbin scale was developed by Cubbin and Jackson in 1991 for ICU patients and revised in 1999 [21,22]. The revised version comprises 12 items: age, weight/tissue viability, past medical history/affecting condition, mental state, mobility, haemodynamics, respiration, oxygen requirement, nutrition, incontinence, and hygiene [22]. Each item is rated from 1 to 4 points. A 1-point deduction from the total score is recommended if the patients fulfil any of the following conditions: requirement of blood products, undergoing surgery, imaging/scan in the past 48 h, or need rewarming due to hypothermia. The maximum score is 40, with lower scores indicating higher risk. The cut-off point for “high risk” was established at 29.

### 2.3. Translation and Cross-Cultural Adaptation

**Figure 1:**
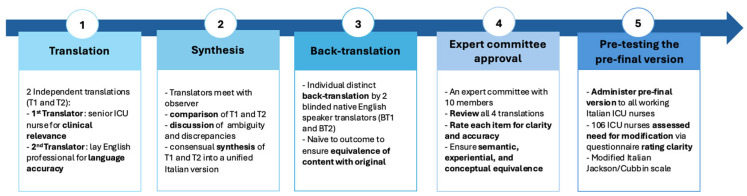
Graphic representation of the stages of the process of translation and cross-cultural adaptation of the Jackson/Cubbin scale into Italian.

A cross-cultural adaptation process was conducted to create the Italian version of the scale. This followed the Guidelines for the Process of Cross-Cultural Adaptation of Self-Report Measures, a widely used and validated guideline for adaptation studies [26]. The methodological steps are described in Figure 1.

*Step 1: Translation*. The first step involved two native Italian translators. The first translator was a senior ICU nurse with ten years of experience and a high level of certification in the English language (C1) to ensure clinical equivalence. The second translator was a lay professional English translator without medical knowledge to produce a version which would reflect good English language construct in Italian. Each translator developed independent Italian versions of the questionnaire (T1 and T2).

*Step 2: Synthesis*. Both translators met with an observer to compare their translations and discuss discrepancies to achieve semantic and syntactic convergence, resulting in a unified version (T-12).

*Step 3: Back-Translation*. Two native English-speaking translators, blinded to the original version, independently back-translated the Italian version. This process ensured equivalence of content with the original questionnaire, resulting in two distinct back-translations (BT1 and BT2).

*Step 4—Expert Committee Approval*. An expert committee composed of ten members was formed to include all key stakeholders recommended by Beaton et al.’s guidelines for translation and cultural adaptation: methodologists, healthcare professionals with ICU expertise, and language professionals (professional translators) [26]. The composition of this committee is crucial to achievement of cross-cultural equivalence. The committee included nurses with specialist higher education qualifications (i.e., a Master’s in Science and/or Postgraduate Certificate in critical care and/or wound care). All members had at least two years of ICU experience, except for one wound care specialist who did not meet this criterion. The committee reviewed all versions (T1, T2, T-12, BT1, BT2) to ensure semantic, idiomatic, experiential, and conceptual equivalence. Each member rated the clarity and accuracy of each item using a 4-point Likert scale (1 = strongly disagree to 4 = strongly agree). Fleiss’ Kappa was calculated to evaluate the inter-rater agreement. According to the interpretation of Fleiss’ Kappa, κ ≤ 0 indicates no agreement, κ between 0.0 and 0.20 slight agreement, κ between 0.21 and 0.40 fair agreement, κ between 0.41 and 0.60 moderate agreement, κ between 0.61 and 0.80 substantial agreement, and κ between 0.81 and 1.0 almost perfect agreement [27]. Upon completion, the pre-final version was prepared for field testing.

*Step 5—Pre-test of the Pre-final Version*. The pre-final version was administered to all ICU nurses working in the two intensive care units at the University Hospital of Modena accounting for 106 participants in total. This approach aimed to include the entire available population of nurses in these settings, ensuring broad representation of ICU nursing staff with varied experience levels. The sample size exceeded the recommended minimum of 30 to 40 participants suggested by Beaton et al. for pre-testing cross-cultural adaptations [26]. Participants were informed about the study, and written informed consent was obtained from all participants prior to enrolment. Each participant completed the questionnaire, reported demographic and professional data, and rated item clarity using a dichotomous scale (clear/unclear). Items rated unclear by ≥20% of participants were revised.

### 2.4. Content Validity

Following the translation and cultural adaptation process, a paper-based questionnaire was administered to ten nurses. These were selected through a stratified convenience sampling method, involving nurses from various intensive care units to ensure representation of different clinical contexts. Each individual expert evaluated all items for relevance, clarity, simplicity, and ambiguity using a 4-point Likert scale, as recommended by Polit et al. [28,29].

An open comments section allowed for additional suggestions. Content validity was determined using the item-level Content Validity Index (I-CVI), scale-level Content Validity Index (S-CVI), and S-CVI-Ave (average method) [28,29].

The number of experts involved in content validity assessment varies in the literature, typically ranging from 3 to 10 reviewers. Lynn et al., 1986 recommends that when fewer than six experts are used, unanimous agreement is necessary to ensure content validity, whereas with more than five experts, some disagreement is acceptable, and an I-CVI threshold of 0.78 or higher is sufficient. We included ten experts to increase the robustness of the evaluation, allowing for a more reliable estimation of item relevance while accommodating potential variability in expert judgments [30].

### 2.5. Data Analysis

All analyses were performed using Excel. Frequencies and percentages were used to describe pilot results. In Step 4, Fleiss’ Kappa was calculated using the online software DATATAB Software (2025). The Content Validity Index was calculated following the recommendations of Polit et al. [29,30].

## 3. Results

### 3.1. Translation and Cross-Cultural Adaptation

During the translation and adaptation process for the Italian context, the research team encountered several challenges. Below are the primary discussion points for each item in the scale.

1. Age

The expert panel identified that in the original scale the age category of 55 years appeared in two indicators. Based on evidence showing increased pressure injury risk with age, the range for the score 2 indicator was adjusted to include ages between 56 and 70 years.

2. Weight/Tissue Viability

Experts debated the translation of “weight” and “tissue viability”. Wound care experts recommended connecting the two terms to highlight the relationship between body weight and tissue viability complications that contribute to pressure injury development. Regarding “tissue viability”, potential translations such as “salubrità”, “floridità”, and “trofismo cutaneo” were discussed. Ultimately, the panel decided to retain a literal translation.

3. PMH—Past Medical History/Affecting Condition

Translating this item posed significant challenges. To ensure cultural adaptation, the panel reviewed the original study’s definition. The authors emphasized that chronic pre-existing conditions may impair tissue viability and worsen with acute illness. The authors also noted the difficulty in exhaustively listing all conditions that affect tissue viability. Consequently, the panel opted to translate the item title as “Condizioni cliniche favorenti” (Conditions promoting pressure injury development), with the following explanatory note: “Comorbidities that promote the development of pressure injuries. For scoring, rely on the nurse’s clinical judgment.”

4. Mental State

The panel debated between “stato mentale” (mental state) and “stato neurologico” (neurological state), ultimately selecting the latter as it is more common in ICU terminology. Additionally, “apathetic” was replaced with “soporific” (drowsy). The term “paralysed” was removed from the score 1 indicator since coma and deep sedation inherently imply immobility. The panel also concluded that tetraplegic patients should fall under the “mobility” item rather than “neurological state”.

5. Mobility

The term “posizione prona” (ready position) was replaced with “posizione obbligata” (compulsory position) to better define positions that cannot be modified due to therapeutic necessity (e.g., prone position in acute respiratory distress syndrome) or severe clinical instability.

6. Inotrope

The panel approved replacing “inotropo” with “vasopressore” (vasopressor), acknowledging that norepinephrine is categorized as a vasopressor and is one of the drugs most strongly linked to pressure injury development [31].

7. Respiration

The panel replaced “CPAP” with “NIV” (non-invasive ventilation) to include all non-invasive ventilation methods and interfaces. The outdated “T-tube” method was eliminated.

8. Oxygen Requirement

The panel revised the T-12 version to make this item more concise while maintaining semantic equivalence.

9. Nutrition

The panel replaced the first indicator with “dieta completa” (complete diet) and retained the literal translation of the final indicator. The panel noted that the Braden scale’s classification system (excellent, adequate, probably inadequate, and very poor) might be preferable. Nevertheless, they agreed to retain the literal translation for this phrase, with potential revisions considered in future research.

10. Incontinence

The term “incontinenza” was replaced with “umidità cutanea” (skin moisture) to better align with the item’s indicators. The first indicator was revised to refer solely to the absence of skin moisture, removing terms such as “anurico/cateterizzato” (anuric/catheterized) since these conditions imply the absence of moisture. Additionally, “umidità cutanea generalizzata” (generalized skin moisture) was introduced to cover a broader range of conditions involving prolonged exposure to various moisture sources across most of the body’s surface.

The overall Fleiss’ Kappa value was 0.74. Fleiss’ Kappa values for each item are reported in Table 1.

### 3.2. Pre-Test of the Pre-Final Version

The version developed at the end of Phase 4 was administered to a total of 106 nurses, achieving a response rate of 83%. The socio-demographic and professional characteristics of the participants are reported in Table 2.

**Figure 2:**
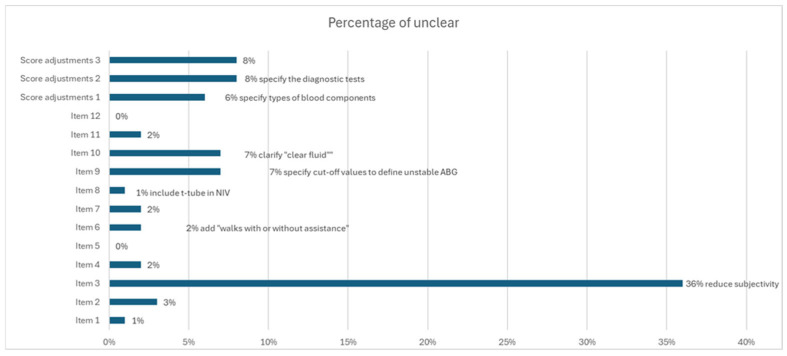
Percentage of unclear items.

The results of the pre-test are reported in Figure 2. Suggested modifications are indicated in relation to specific items, where applicable.

Item 3 was the only item that did not meet the predetermined clarity threshold of 80%, as more than 20% of respondents found it unclear. The main reason accounting for this was the perceived subjectivity in assessing the severity of comorbidities in relation to the patient’s primary diagnosis. Several nurses reported difficulties in interpreting the item consistently, especially in the absence of precise clinical examples or objective criteria.

To address this issue, two main solutions were proposed:

- To include, under each scoring level, a list of comorbid conditions that promote the development of pressure injuries.
- To assign a specific number of comorbidities to each scoring level in order to reduce interpretation variability.

These proposals aim to improve clarity and inter-rater reliability by reducing the reliance on individual clinical judgment alone.

All other items exceeded the 80% clarity threshold; however, several nurses suggested minor modifications to improve comprehension. For item 9, some participants proposed to include specific cut-off values to define what constitutes “unstable arterial blood gases” in order to reduce ambiguity. One nurse also recommended further simplifying the item.

Additional concerns were raised regarding item 10, which had already been discussed during the expert panel review. Specifically, nurses requested clearer definitions of the term “clear fluids” and better categorization of hypertonic glucose solutions (e.g., 33%, 50%, or higher concentrations).

### 3.3. Content Validity

The socio-demographic and professional characteristics of the nurses who participated in this phase are described in Table 3. The ten nurses invited to participate in this phase worked in intensive care units in various Italian cities (i.e., Bologna, Verona, Modena, Bari, and Bolzano) to enhance the generalizability of the results.

Data related to the “relevance” domain were used for the calculation of the Content Validity Index (CVI). Data concerning “clarity”, “simplicity”, and “ambiguity” were utilized to support the findings obtained previously in Phase 5 (Pre-Test of the Pre-final version). Table 4 presents a summary of the results obtained, detailing the number of experts who assigned each score.

The I-CVI for each item is reported in Table 4. For the calculation of the S-CVI, only the 12 items of the scale were considered. The resulting S-CVI was 0.92. Additionally, the S-CVI-Ave, representing the average of the S-CVI scores assigned by the experts for all scale items, was 0.94.

## 4. Discussion

The Italian Society of Anaesthesia, Analgesia, Resuscitation, and Intensive Care (SIAARTI) emphasizes the importance of preventive strategies in reducing the risk of pressure injuries among critically ill patients. Within these strategies, the use of validated risk assessment scales is a key component of evidence-based clinical practice [32]. However, despite their documented effectiveness, there is limited information regarding the extent to which these tools are routinely adopted and systematically integrated into clinical workflows, particularly in intensive care settings. Moreover, many of the most widely used assessment scales were originally developed and validated in English, which may pose a significant language barrier to effective implementation in non-English-speaking countries such as Italy. This language-related limitation can impede access to adequate training and compromise the correct use of the tools, potentially affecting the consistency and overall quality of pressure injury prevention practices [33].

The Jackson/Cubbin scale, like many other nursing assessment instruments, was originally developed and validated in English [21,22]. For Italian nurses to effectively implement such tools, a thorough validation process involving both language and content experts is essential. In line with established international guidelines, we conducted a study aimed at the translation and cultural adaptation of the Jackson/Cubbin scale [26]. This process resulted in an Italian version that is conceptually equivalent and linguistically appropriate. Nevertheless, cross-cultural adaptation may introduce distortions: while linguistic accuracy can be achieved, cultural relevance is not always ensured [33].

This issue was particularly evident in our study, which resulted in a linguistically sound version of the scale but required partial content adjustments, most likely because the original instrument was developed in 1991. During the clarity evaluation phase, participating nurses highlighted two main needs: (1) to update the scale based on current evidence, and (2) to enhance the objectivity of item indicators to reduce inter-rater variability. To address these concerns, the expert panel recommended possible specific modifications to selected items to improve consistency and applicability in clinical practice.

These changes involved not only linguistic or cultural adaptation but also a partial conceptual transformation of the original instrument. As such, the Italian version should be clearly positioned as a “modified Italian version” of the Jackson/Cubbin scale. While these modifications may enhance clinical relevance in the Italian ICU context, they may also limit direct comparability with the original scale and potentially affect construct equivalence.

Despite item 2 (Weight) and item 9 (Nutrition) having only moderate agreement (according to Fleiss’ Kappa values), they were intentionally left unmodified to maintain a more literal translation during the content validation phase. These items may be revised in future validation studies, and we offer below item-by-item suggestions and recommendations for future modification of the instrument.

Item 1—Age

Multiple studies have demonstrated that increasing age is associated with a higher risk of pressure injuries. Specifically, several multivariate analyses have shown that individuals aged 70 years or older face an increased risk of developing pressure injuries [1]. Some experts suggested using an absolute value or narrowing the range intervals.

Item 2—Weight

Experts recommended replacing weight with Body Mass Index (BMI) and revising the corresponding indicators. This change would improve objectivity in completing this item. This adjustment was not implemented in the current version but may be considered in future revisions.

Item 3—PMH—Past Medical History/Affecting Condition

This item proved to be the most problematic, with 36% of the nurses in Phase 5 finding it unclear. This concern was echoed by 40% of the experts who rated the item as “not entirely clear”, with an additional 20% rating it as “fairly clear but requiring revision”. Furthermore, 40% of the experts deemed the item “ambiguous”. The item was felt to be unclear or ambiguous due to being too generic and potentially leading to misinterpretation. To improve clarity, nurses suggested listing specific clinical conditions for each indicator or assigning a set number of comorbidities for each indicator. Combining both suggestions may be the most suitable solution, as the original authors emphasized the difficulty of exhaustively identifying all comorbidities that increase pressure injury risk and classifying them under a single indicator.

Item 5—Mobility

While 40% of the experts involved in content validation rated this item as “fairly clear and simple but requiring revision”, all nurses in Phase 5 found it clear. Some experts suggested incorporating the Richmond Agitation–Sedation Scale (RASS) to improve the objectivity of the indicators.

Item 8—Oxygen Requirement

Experts proposed replacing “dyspnoea at rest” with “tachypnoeic” to make the indicator more objective.

Item 9—Nutrition

Experts requested measures to reduce inter-rater variability for this item, including defining specific arterial blood gas values to identify instability and providing defined desaturation ranges during mobilization.

The results from the Content Validity Index (CVI) calculations confirmed that the scale demonstrated good content validity, having exceeded the required cut-off values (i.e., ≥0.78). However, given the partial conceptual transformation, further psychometric testing is warranted to fully validate the instrument in its modified form. Specifically, further studies should be conducted to assess its construct validity, predictive validity, factor structure, and inter-rater reliability.

In conclusion, this study represents an important first step toward the psychometric validation of the Jackson/Cubbin scale in the Italian landscape, providing a linguistically and conceptually adapted version suitable for use in intensive care settings. Further research is needed to establish its validity and reliability as a distinct instrument within the Italian context.

## 5. Strengths and Limitations

This study enabled the translation of the Jackson/Cubbin scale for its implementation in the Italian context, in accordance with the recommendations of the Italian Society of Anaesthesia, Analgesia, Resuscitation, and Intensive Care (SIAARTI) [32]. The study, however, presents some limitations. Firstly, we used a convenience sample of nurses across two centres. To minimize bias and strengthen content validity, we drew experts from various ICUs across different Italian cities to improve generalizability.

A second important limitation of this study concerns the inability to obtain formal authorization from the original authors of the scale, despite repeated attempts to contact them through various communication channels. This limited the possibility of directly comparing our version with any existing official translations and receiving specific guidance from the authors on the cultural adaptation process. Furthermore, this may also raise ethical and legal concerns regarding the future dissemination and implementation of the Italian version. Although we strictly followed international guidelines for the translation and validation of psychometric instruments (including the back-translation process and expert review), the absence of direct consultation with the original authors may have influenced certain aspects of the conceptual and semantic equivalence of the scale. However, the involvement of a panel of experts with strong clinical and methodological expertise contributed to ensuring high methodological quality, rigor, and validity during the adaptation process.

Moreover, some of the changes made during the adaptation process may reflect not only linguistic or cultural adjustments but also a partial conceptual restructuring of the original scale. These modifications were deemed necessary to improve clarity, clinical applicability, and alignment with current practice in Italian ICUs. While such adjustments may enhance the scale’s relevance and usability in the local context, they may also limit direct comparability with the original version and potentially compromise construct equivalence.

Therefore, this version should be clearly presented as a modified Italian version of the Jackson/Cubbin scale. Further studies are needed to evaluate its construct and predictive validity, inter-rater reliability, and overall psychometric properties to confirm its robustness as an assessment tool in the Italian intensive care context.

## 6. Conclusions

This cross-cultural adaptation study resulted in a linguistically accurate and conceptually modified Jackson/Cubbin scale with good content validity, highlighting potential item modifications to reflect current evidence and reduce inter-rater variability. This work represents a first step toward the implementation of the modified Jackson/Cubbin scale in Italian clinical ICU settings. Translation into the local language is essential to ensure adequate implementation of risk-assessment scales for pressure injuries in clinical practice. Further research is needed to refine the scale and adapt it further to the needs of ICU nurses. Future studies may focus on testing its construct and predictive validity, inter-rater reliability, and factor structure analysis. Additionally, it will be important to evaluate its application in the ICU setting to identify potential clinical issues and additional barriers to its effective implementation.

## Figures and Tables

**Table 1:** Fleiss’ Kappa results for each item on the scale.

Item	Fleiss’ κ	Interpretation
**1**	0.78	Substantial agreement
**2**	0.69	Moderate agreement
**3**	0.80	Substantial agreement
**4**	0.73	Substantial agreement
**5**	0.76	Substantial agreement
**6**	0.71	Substantial agreement
**7**	0.72	Substantial agreement
**8**	0.75	Substantial agreement
**9**	0.68	Moderate agreement
**10**	0.77	Substantial agreement
**11**	0.70	Substantial agreement
**12**	0.74	Substantial agreement

**Table 2:** Characteristics of the participants in the pre-test phase.

Characteristics of the Study Participants	
Gender (N) %	
Male	(65) 74%
Female	(23) 26%
Age, years (N) %	
22–34	(41) 47%
35–45	(29) 33%
46–60	(14) 16%
Over 60	(4) 4%
Education Level (N) %	
Diploma	(6) 7%
Bachelor’s degree	(54) 61%
Bachelor’s degree and Postgraduate Certificate in Critical Care	(16) 18%
Bachelor’s degree and other postgraduate certificate qualification	(9) 10%
Master’s degree in science	(1) 1%
Master’s degree in science and Postgraduate Certificate in Critical Care	(1) 1%
Not declared	(1) 1%
Years of experience (mean ± SD)	14.14 ± 9.07
Years of experience in ICU (mean ± SD)	10.7 ± 8.83

**Table 3:** Demographic characteristics of participants in the CVI phase.

Characteristics of the Study Participants	
Gender (N) %	
Male	(7) 70%
Female	(3) 30%
Age, years (N) %	
22–34	(5) 50%
35–45	(4) 40%
46–60	(1) 10%
Education Level (N) %	
Bachelor’s degree and Postgraduate Certificate in Critical Care	(5) 50%
Master’s degree in science and Postgraduate Certificate in Critical Care	(5) 50%
Years of experience (mean ± SD)	9.9 ± 7.1
Years of experience in ICU (mean ± SD)	8 ± 6.6

**Table 4:** Evaluation of the Italian-translated Jackson/Cubbin scale items: relevance, simplicity, clarity, ambiguity, and I-CVI.

Item	Relevance				Clarity				Simplicity				Ambiguity				I-CVI
	***1***	***2***	***3***	***4***	***1***	***2***	***3***	***4***	***1***	***2***	***3***	***4***	***1***	***2***	***3***	***4***	
Age	0	2	4	4	0	0	3	7	0	0	2	8	0	0	0	10	**0.8**
Weight/Tissue Viability	0	0	2	8	2	1	2	5	2	3	0	5	2	2	1	5	**1**
PMH/Affecting Condition	0	0	2	8	4	2	2	2	3	3	2	2	4	1	3	2	**1**
General Skin Condition	0	0	3	7	0	0	4	6	0	1	6	3	0	0	5	5	**1**
Mental State	0	0	2	8	0	5	1	4	0	5	1	4	0	2	4	4	**1**
Mobility	0	0	1	9	0	3	0	7	0	1	2	7	0	1	3	6	**1**
Haemodynamics	0	0	5	5	0	2	3	5	0	1	4	5	0	0	4	6	**1**
Respiration	0	1	3	6	0	4	1	5	0	2	4	4	0	3	2	5	**0.9**
Oxygen Requirements	0	1	3	6	0	3	4	3	0	2	5	3	1	1	3	5	**0.9**
Nutrition	0	1	3	6	1	4	1	4	0	0	3	7	0	1	5	4	**0.9**
Incontinence	0	1	0	9	0	2	2	6	0	2	3	5	0	1	4	5	**0.9**
Hygiene	0	1	0	9	0	0	1	9	0	0	1	9	0	0	1	9	**0.9**
Deduct 1 point → time spent in surgery/scan in last 48 h	0	0	0	10	1	2	1	7	0	2	0	8	0	0	2	8	**1**
Deduct 1 point → if requires blood products	0	0	1	9	0	0	1	9	0	0	1	9	0	0	1	9	**1**
Deduct 1 point → for hypothermia until warm	0	0	1	9	0	0	0	10	0	0	0	10	0	0	1	9	**1**

## Data Availability

Data are contained within the article and Supplementary Materials.

## Supplementary Materials

The following supporting information can be downloaded at: https://www.mdpi.com/article/10.3390/nursrep15070256/s1, Table S1. Modified Italian Version of the Jackson/Cubbin Pressure Injury Risk Assessment Scale.

## Public Involvement Statement

Please describe how the public (patients, consumers, carers) were involved in the research. Consider reporting against the GRIPP2 (Guidance for Reporting Involvement of Patients and the Public) checklist. If the public were not involved in any aspect of the research add: “No public involvement in any aspect of this research”.

## Guidelines and Standards Statement

We followed the STROBE checklist to ensure clear and transparent reporting of the observational study findings.

## Abbreviations

ICU	Intensive Care Unit
CVI	Content Validity Index
S-CVI	Scale-level Content Validity Index
