# Supplementary material for: Translation, Cultural Adaptation, and Content Validity of a Modified Italian Version of the Jackson/Cubbin Pressure Injury Risk Assessment Scale for ICU Patients
Source: Nurs Rep. 2025 Jul 14;15(7):256. doi: 10.3390/nursrep15070256 (PMC12300118; doi:10.3390/nursrep15070256)
Supplement: Supplementary file 1 [file nursrep-15-00256-s001.zip › nursrep-3683397-supplementary.pdf]

Modified Italian Version of the Jackson/Cubbin Pressure Injury Risk Assessment Scale

**Età**		**Peso e vitalità tissutale**		**Condizioni cliniche favorenti**<br>*Comorbilità che favoriscono lo sviluppo di lesioni da pressione. Per assegnare il punteggio fare affidamento sul giudizio clinico dell'infermiere.*		**Condizioni generali della cute**		**Stato neurologico**		**Mobilità**	
<40	4	Normopeso	4	Nessuna	4	Cute integra	4	Cosciente e vigile	4	Cammina con aiuto	4
40-45	3	Obeso	3	Lievi	3	Cute/aree arrossate (a rischio di lesione)	3	Agitato/irrequieto/confuso	3	Molto limitata/costretto su sedia a rotelle	3
56-70	2	Cachettico	2	Severe	2	Abrasione/cute escoriata (superficiale)	2	Soporoso/sedato ma responsivo	2	Immobile ma tollera cambi di posizione	2
>870	1	Uno dei precedenti + edema	1	Molto severe	1	Necrosi /lesione essudante (profonda)	1	Coma/non responsivo/sedato	1	Non tollera cambi di posizione/postura obbligata	1
**Emodinamica**		**Respirazione**		**Necessità di ossigeno**		**Nutrizione**		**Umidità cutanea**		**Igiene**	
Stabile senza vasopressori	4	Spontanea	4	FiO_2 < 40%, stabile durante i movimenti	4	Dieta completa	4	Assente	4	Indipendente	4
Stabile con vasopressori 3	3	NIV	3	FiO_2 dal 40 % al 60%, stabile durante i movimenti	3	Dieta leggera, nutrizione enterale	3	Incontinenza urinaria/ umidità cutanea generalizzata	3	Bisogno di assistenza minima	3
Instabile senza vasopressori 2	2	Ventilazione meccanica invasiva	2	FiO_2 dal 40 % al 60%, EGA stabile ma desaturazione durante i movimenti	2	Nutrizione parenterale	2	Incontinenza fecale/diarrea occasionale	2	Bisogno di assistenza elevata	2
Instabile con vasopressori	1	Dispnea a riposo	1	FiO_2> 60%, EGA instabile, desaturazione a riposo	1	Solo liquidi chiari per via endovenosa	1	Incontinenza urinaria e fecale/diarrea prolungata	1	Completamente dipendente	1

Detrarre 1 punto → se sottoposto ad intervento chirurgico o ad indagini diagnostiche nelle ultime 48 ore
Detrarre 1 punto →se necessita di emocomponenti
Detrarre 1 punto →in caso di ipotermia
Possibile punteggio 48/48. Alto rischio → se punteggio 29/48 o al di sotto
